# Removal of External Tumors

**Published:** 1873-04

**Authors:** J. E. Nichols

**Affiliations:** Osage, Mitchell Co., Iowa


ARTICLE II.—*Removal of External Tumors.* By J. E. NICHOLS, M.D., Osage, Mitchell Co., Iowa.

On page 509 of the last volume of the JOURNAL there is an account of Michel's process for removing external tumors.

Determine as we may, the most dignified of our profession find human nature too weak to avoid an occasional step in the direction of charlatan practice. Without precisely knowing why we do certain things, we will launch out into unknown depths and make a trial of something with the most sanguine expectations.

With this preface as my only excuse, let me narrate a process I have employed the last year for the removal of external tumors with unlooked-for success. I will first state that the process is not original with me; but rather of the order charlatan. I will proceed at once to the cases in contemplation.

Feb. 6, 1872. Mrs. B—, aged about twenty years, applied to me for the relief of a dry, hard and very painful tumor in the palm of the right hand. It was then about an inch long and eight lines broad, nearly level with the skin, slightly bluish in color, dry and squamous on the surface. Had been forming over two years, and when first noticed was about the size of a pin head.

She had visited another doctor before coming to me, and he had pronounced it a cancer, and desired to remove it by extirpation in the usual manner. I sanctioned his diagnosis and advised the same treatment; but she declared she would die before she would have it "cut out," and desired to know if there was not some kind of salve that would remove it. She had heard of such measures being used. Following the hints of more learned surgeons, I replied, that plasters, I believed, were sometimes used but with indifferent success, and with almost certain recurrence of the difficulty. Imagine my very embarrassed position when she desired me to use any of the plasters referred to, and that she would abide the result, no matter what it might be. I never had heard of but one "cancer plaster," but I at once resolved to test the qualities of this. I took—

R. Chloride Zinc, (granular), } *aa*, q. s.,

Pulv. Sanguin. Radix, }

To make enough paste to cover the tumor, and rubbed the two together in the open air until deliquescence occurred, forming a very smooth, firm paste. I then cut out a hole in a piece of common adhesive plaster to correspond to the size and shape of the tumor, and stuck it on to protect the sound tissues from my application. Then spreading the paste evenly over the whole tumor, to the depth of half a line, I put over the whole another piece of adhesive plaster, not only to keep the paste in position but to get the thing out of sight, and bade my patient let it remain so for forty-eight hours, when I would again examine it.

Feb. 8. Removed the adhesive plasters, and was very agreeably surprised to find the tumor presenting a parched and withered appearance, and separated by a narrow, shallow seam from the healthy substance in nearly its whole circumference. I ordered a dressing of simple cerate after thoroughly cleansing it with soap and water.

Feb. 10. The simple cerate dressing has been changed twice a day. To-day the tumor seems to be entirely separated, by suppuration, from the sound parts, and lies in the wound like a foreign body. Taking it up carefully with dressing forceps I discovered several fine filaments reaching down into the bottom of the cavity, and as the slightest tension produced pain, I allowed it to remain.

Feb. 11. To-day I succeeded in picking out the tumor without the slightest twinge of pain. Ordered the simple cerate dressing, and in a few days the wound was completely filled with granulations and healed over. She declared that with the exception of a few hours' slight pain and lameness in the hand, she had not suffered at all from the removal. The tumor when out resembled a scorched piece of leather half an inch thick and of the dimensions before given.

March 3, 1872. She again consulted me, and exhibiting two little points in the cicatrix the size of a pin head, and quite red, informed me that they pained her considerably, and desired the plaster to be again applied to them. Fitting the adhesive plaster around them, as with the larger one, I applied the merest particle of paste to each, and in a few days had the satisfaction of removing them, each the size of duck shot. I have seen her repeatedly up to this date, but there has been no recurrence of the tumor or pain.

Soon after this, Mrs. A., aged about 50 years, consulted me about a tumor under the right eye, half or three-quarters of an inch from the lower border of the orbit, and about the same distance from the nose. It was quite red and painful, and had been three years in forming. I used the paste in precisely the same manner as before.

In forty-eight hours I removed the plasters and found considerable inflammation, but no tendency to separate from the healthy parts. Applied the plaster around it, and used the paste again. Forty-eight hours afterwards I removed the plasters, and as she lived some distance from my office, I told her to apply the simple cerate till the tumor was loose or came out of its own accord, and then continue the same dressing till it healed.

Aug. 26, 1872. She came to my office with a red, protruding tumor twice the size of the first. Said that the old one came out all right, and the wound healed up, but that this one soon began to grow. She had been careless about having it removed until now it was too painful to endure.

It was easily removed with one application of the paste, and the after-dressing of simple cerate. It has not yet recurred.

Nov. 10, 1870. Mrs. K— came to me with what I diagnosed, after Erichsen, as an encysted abscess of the right breast. Treated it with tr. iodine, etc., till Dec. 18, when I administered chloroform, and inserted two setons. Ordered stimulating applications topically, and some alterative treatment.

July 19, 1871. The breast seems to be all right; the setons were removed, and the abscess perfectly cicatrized.

March 20. Appears with a new abscess directly over the site of the old one. Will not submit to setons. Is opposed to the exploring needle, and positively objects to the lance.

R. Cut a hole in a piece of emplastrum adhesivi the size of a hazlenut, and placed it over the most prominent part of the abscess. Applied the paste.

In four days, I removed a plug of destroyed tissue. Put a very small quantity of the paste in the opening thus made, filled in with a tampon of cotton-wool, and in two days' time was pleased to learn that large quantities of pus had escaped from the abscess. I now ordered the simple cerate dressing.

April 20. It is healed up and the mammary gland natural, except the cicatrices. Has not recurred.

I now began to conceive that so far as removing external tumors was concerned I was possessed of something as potent as Aladdin's lamp, and for the next six months not a tumor, wart, or excrescence of any kind, came to my notice, but what an immediate application of the "Cancer Paste" proved as efficacious as the barber's salve did to the humpback's neck in removing the fishbone. At last my eyes were opened to the necessity of caution in the administration of this all-potent and quackish paste, by accidentally causing a fistulous abscess in the parotid gland in removing an obstinate tumor (simple, however,) of the neck. But whether its removal by knife might not have been more disastrous I am not prepared to say. I am now operating on a tumor of the antrum of Highmore in the case of a young man who came before our medical association last fall. It was the general verdict that the right superior maxillary must be extirpated. I have repeatedly applied the paste on cotton, to the cavity formed by the expulsion of the bicuspid, canine and two molar teeth. The tumor has gradually come away in large pieces, and smaller ones, and the tumor lessened in size more than one-half at this date. I will try to remember and report the result when the treatment is completed.

On the whole, I conclude that this paste is useful in removing cutaneous tumors where the patient will not yield to the knife; and that for the removal of cancers it is less painful and more successful than extirpation in the ordinary way. I will, however, advise care in its use, that too great inflammation may not occur from applying it to a large surface at a time. If you have a large tumor, remove it by repeated applications through a small opening in the integument made by the first application. Whether it rests with charlatans to make use of such agents depends upon the patience and good nature of our Regulars. Of one thing I am wholly convinced by a continued observation of years, and that is, that many a good thing is poh-pohed at by those who ought to look into the matter and determine, by actual experiment, whether it is good or otherwise.

Further, allow me to say, in conclusion, that if the whole system of a cancerous patient seems to be impregnated with the malady, and the cancerous cachexia is everywhere visible, this means, as well as all others, is futile. A cancer should be removed ere it is fully known to be a cancer, and the use of the "Cancer Paste" will often gain the consent of patients for an early operation when they would not grant it otherwise.

